# A Veteran-Centric Web-Based Decision Aid for Lung Cancer Screening: Usability Analysis

**DOI:** 10.2196/29039

**Published:** 2022-04-08

**Authors:** Marilyn M Schapira, Sumedha Chhatre, Jason M Prigge, Jessica Meline, Dana Kaminstein, Keri L Rodriguez, Liana Fraenkel, Jeffrey D Kravetz, Jeff Whittle, Lori A Bastian, Anil Vachani, Scott Akers, Susan Schrand, Jennifer V Ibarra, Onur Asan

**Affiliations:** 1 Center for Health Equity Research and Promotion Michael J Crescenz VA Medical Center Philadelphia, PA United States; 2 Department of Medicine, University of Pennsylvania School of Medicine Philadelphia, PA United States; 3 Department of Psychiatry, University of Pennsylvania School of Medicine Philadelphia, PA United States; 4 Department of Organizational Dynamics School of Arts & Sciences University of Pennsylvania Phlladelphia, PA United States; 5 Center for Health Equity Research and Promotion VA Pittsburgh Healthcare System Pittsburgh, PA United States; 6 Department of Rheumatology, Berkshire Health Systems Pittsfield, MA United States; 7 Department of Medicine, Yale University New Haven, CT United States; 8 VA Connecticut Healthcare System West Haven, CT United States; 9 Clement J Zablocki VA Medical Center Milwaukee, WI United States; 10 Department of Medicine, Michael J Crescenz VA Medical Center Philadelphia, PA United States; 11 Division of Pulmonary, Allergy, and Critical Care, Department of Medicine, University of Pennsylvania School of Medicine Philadelphia, PA United States; 12 Department of Radiology, Michael J Crescenz VA Medical Center Philadelphia, PA United States; 13 Department of Radiology, University of Pennsylvania School of Medicine Philadelphia, PA United States; 14 The Stevens Institute of Technology, School of Systems and Enterprise Hoboken, NJ United States

**Keywords:** lung cancer screening, decision aid, usability, implementation, cancer screening, shared decision-making, veterans, patient engagement, mobile phone

## Abstract

**Background:**

Web-based tools developed to facilitate a shared decision-making (SDM) process may facilitate the implementation of lung cancer screening (LCS), an evidence-based intervention to improve cancer outcomes. Veterans have specific risk factors and shared experiences that affect the benefits and potential harms of LCS and thus may value a veteran-centric LCS decision tool (LCSDecTool).

**Objective:**

This study aims to conduct usability testing of an LCSDecTool designed for veterans receiving care at a Veteran Affairs medical center.

**Methods:**

Usability testing of the LCSDecTool was conducted in a prototype version (phase 1) and a high-fidelity version (phase 2). A total of 18 veterans and 8 clinicians participated in phase 1, and 43 veterans participated in phase 2. Quantitative outcomes from the users included the System Usability Scale (SUS) and the End User Computing Satisfaction (EUCS) in phase 1 and the SUS, EUCS, and Patient Engagement scale in phase 2. Qualitative data were obtained from observations of user sessions and brief interviews. The results of phase 1 informed the modifications of the prototype for the high-fidelity version. Phase 2 usability testing took place in the context of a pilot hybrid type 1 effectiveness-implementation trial.

**Results:**

In the phase 1 prototype usability testing, the mean SUS score (potential range: 0-100) was 81.90 (SD 9.80), corresponding to an excellent level of usability. The mean EUCS score (potential range: 1-5) was 4.30 (SD 0.71). In the phase 2 high-fidelity usability testing, the mean SUS score was 65.76 (SD 15.23), corresponding to a good level of usability. The mean EUCS score was 3.91 (SD 0.95); and the mean Patient Engagement scale score (potential range 1 [low] to 5 [high]) was 4.62 (SD 0.67). The median time to completion in minutes was 13 (IQR 10-16). A thematic analysis of user statements documented during phase 2 high-fidelity usability testing identified the following themes: a low baseline level of awareness and knowledge about LCS increased after use of the LCSDecTool; users sought more detailed descriptions about the LCS process; the LCSDecTool was generally easy to use, but specific navigation challenges remained; some users noted difficulty understanding medical terms used in the LCSDecTool; and use of the tool evoked veterans’ struggles with prior attempts at smoking cessation.

**Conclusions:**

Our findings support the development and use of this eHealth technology in the primary care clinical setting as a way to engage veterans, inform them about a new cancer control screening test, and prepare them to participate in an SDM discussion with their provider.

## Introduction

### Background

Shared decision-making (SDM) is a valuable and, in some settings, mandated approach to helping patients make informed and value-aligned decisions in medical care. SDM is especially useful in decisions of equipoise when the benefits of intervention do not clearly outweigh the harms and when the value patients assign to these attributes vary [1,2]. Lung cancer screening (LCS) is an evidence-based intervention that meets these criteria [3,4]. However, despite expert guidelines dating from 2014 that recommend LCS for eligible persons [5], LCS uptake and adherence to follow-up protocols have remained low [6]. A number of SDM tools for LCS have been developed [7-10]; however, the implementation of SDM in practice has been limited [11], and the uptake of LCS remains low [12]. A participatory design approach, including patient end users such as US veterans, may increase the uptake of SDM tools and support value-aligned decisions regarding LCS. A user-centered design of SDM interventions guided by usability metrics is required to advance the integration of web-based SDM tools into clinical practice [13].

LCS with low-dose computed tomography has proven effective in decreasing lung cancer mortality in two large randomized controlled trials: the National Lung Screening Trial in the United States [4] and, more recently, the Dutch–Belgian LCS trial [3]. The trials reported a decrease of 20% and 25% in lung cancer mortality among those screened compared with control populations for the United States and Belgian studies, respectively. However, clinical trials also reported harms, including false positive tests, significant incidental findings, and an excess incidence of lung cancer cases indicative of overdiagnosis. False positive tests require follow-up imaging and, in some cases, invasive diagnostic procedures that can cause harm. False positive rates vary across trials and decrease in the later years of screening. The National Lung Screening Trial reported false positive rates of 26.3 %, 27.2 %, and 15.9% at baseline, year 1, and year 2, respectively. The Dutch–Belgian trial reported false positive rates of 19.8 %, 7.1 %, and 9.0% at baseline, year 1, and year 3, respectively [14].

Decision aids (DAs) are structured tools that include a description of decision options, evidence-based benefits and harms associated with each option, a value clarification exercise, and support for a SDM conversation with one’s health care provider [15]. In systematic reviews, DAs have been found to improve the quality of decision-making with respect to the outcomes of knowledge, preparation for decision-making, and value-aligned decisions [16]. However, the implementation of DAs has been limited. A primary barrier has been their integration into the flow of clinical practice [17-19].

### Objectives

Veterans of the United States military are at a higher risk of developing lung cancer than the general population because of older age, higher rates of smoking, and environmental exposures [20]. Veterans are also at higher risk for mental health conditions, including anxiety and posttraumatic stress disorder (PTSD), which increase the burden of LCS, thus affecting the balance of benefits and potential harm from LCS [20]. To address these challenges, we used a user-centered design to develop a veteran-centric LCS decision tool (LCSDecTool) for use in a primary care setting. This tool was designed to be used independently by the patient before the clinic visit (either at home or in the waiting room) with the option of sharing some components with the clinician during the clinic visit. In this study, we report an iterative process of usability testing of a prototype tool and a revised high-fidelity version, with the latter conducted as part of a hybrid type 1 effectiveness-implementation trial. Usability assessment is a key component in the development of web-based decision support tools. Common methods used in usability testing include observation, cognitive interviews, and self-reported feasibility and usability with validated scales [21]. We seek to determine whether veterans find the tool useful to use in the context of a clinical visit and elucidate key aspects of the user experience that had an impact on usability.

## Methods

### Description of the LCSDecTool

We developed the LCSDecTool using the Promoting Action on Research Implementation in Health Services (PARiHS) implementation framework. The PARiHS framework includes the domains of evidence (scientific evidence supporting intervention efficacy), context (the setting in which the intervention is delivered), and facilitation (training and support needed to deliver the intervention) [22,23]. Systematic reviews have established the efficacy of decision aids in improving the process of medical decision-making, as indicated by an increase in knowledge, perceptions of being informed, accuracy of risk perceptions, and clarity about values [24]. We were guided by criteria from the PARiHS framework in the tool design and usability testing for the prototype and high-fidelity LCSDecTool, both of which were evaluated among eligible veterans receiving primary care in Veteran Affairs (VA) Medical Centers and later in the context of a clinical visit. Both focused on the support needed by veterans to access and navigate the tool. The development and usability assessment were also informed by the Technology Acceptance Model (TAM) [25,26]. The TAM posits that people need to perceive the technology as useful and easy to use to continue using it. This model supported our choice of the quantitative outcome measures of the System Usability Scale (SUS) and the End User Computing Satisfaction (EUCS) measure, which include questions in these domains [27-29].

Stakeholder focus groups (clinicians) and structured interviews (veterans) further informed the content and features of the prototype LCSDecTool [30]. The LCSDecTool was designed to (1) use in advance of or during a primary care clinical visit where LCS may be initiated, (2) ensure that veterans understand the key benefits and potential harms of LCS, (3) help veterans weigh the benefits and potential harms of LCS, (4) provide resources for smoking cessation and mental health treatment, (5) support communication with their provider regarding this decision, and (6) include a clinician portal to streamline the use of the tool with a clinician in the clinical setting. The tool was designed for the primary platform of a tablet but has compatibility with a computer or mobile phone. The stakeholder inputs that informed these goals are summarized in Table 1. The data collection form with illustrations from the prototype version of the LCSDecTool is available in the supplemental materials (Multimedia Appendix 1).

**Table 1:** Feedback from veteran and clinician stakeholders to support the design of the lung cancer screening shared decision-making tool.

Features and content and description			Supportive data from stakeholder interviews		
			Veterans (n=32)		Clinicians (n=9)
**Computer based**					
	Accessed by a URL link on devices: tablet, desktop, laptop, and smartphone	12 (38%) recommended the lung cancer screening shared decision-making tool be computer based; some wanted paper instead of digital		6 (67%) supported a web-based module 5 (56%) supported a phone app 4 (44%) supported an app for a tablet	
**Overview of LCS^a^: simulated discussion between patient and provider**					
	Simulated dialog with questions and answers about LCS	8 (25%) recommend a web-based tool that is engaging and interactive to hold attention; one suggested that they could ask a question, and it would be answered back		Only 2 (22%) indicated they thought patients were knowledgeable about LCS	
**Overview of LCS: clickable knowledge boxes**					
	6 knowledge boxes, each covering a key LCS content area; must click on all boxes before advancing to the tool	17 (53%) recommended a simple user-friendly website, suggested simple words, examples, and graphs; break knowledge down into topic categories, and have limited words on each page		Only 2 (22%) indicated they thought patients were knowledgeable about LCS	
**Evidence-based outcomes: pictograph**					
	Main outcomes from the National Lung Screening Trial displayed in pictograph: lung cancer deaths and deaths averted, false positives, biopsies, and complications	17 (53%) commented they wanted understandable graphics 10 (31%) commented they wanted updates in research		4 (44%) wanted graphical representation of risks and benefits	
**Value elicitation—rating scale 1 and rating scale 2 and Cancer Screening Attitudes Rating Scale**					
	Ratings to indicate value attributed to LDCT^b^ attributes; high-fidelity version used rating scale 2	Qualitative analysis of 23 (72%) veteran interviews indicates wide variation in how LCS attributes are valued and that attitude and beliefs about LCS may affect value ratings [30]		4 (44%) noted that LCS is not a priority for veterans compared with their other concerns	
**Veteran-centric content—smoking cessation and mental health**					
	VA^c^ resources highlighted with an option to request consultation	12 (38%) wanted to include smoking cessation options in the tool 19 (59%) indicated that LCS might increase their anxiety or worry		6 (67%) wanted to use LCS discussions to promote smoking cessation 4 (44%) noted that LCS might increase patient anxiety and worry about having cancer	
**Enter questions for the provider**					
	Free text option; questions inserted on the summary sheet	8 (25%) stated that they wanted the tool to be engaging and interactive		—^d^	
**Summary page**					
	Includes ratings of values and attitudes; ability to print, save, or email page	10 (31%) veterans stated the tool should prepare them for a discussion about LCS with their provider		—	
**Clinician portal**					
	Link from entry page to features for use at the point of care: pictograph, value and attitude assessment, and summary page	—		5 (56%) noted they did not have time to participate in shared decision-making about LCS. Clinicians indicated that the tool must be short and easy to use in the clinical setting.	

^a^LCS: lung cancer screening.

^b^LDCT: low-dose computed tomography.

^c^VA: Veteran Affairs.

^d^Data not available.

### Approach to Usability Testing

We used both quantitative and qualitative methods for usability testing. The SUS and the EUCS scales have been widely used and well-validated to assess interventions in the domains of ease of use and usefulness [28,29,31-36]. The SUS is a 10-item scale developed for the assessment of a broad range of products; scores (ranging from 0=low usability to 100=high usability) correspond to the following adjective descriptors: worst imaginable, awful, poor, okay, good, excellent, and best imaginable [27,37]. The EUCS scale is a 12-item scale that captures the domains of content, accuracy, format, ease of use, and timeliness relevant to a computer application. Scores range from 1=low satisfaction to 5=high satisfaction [28,29]. The Patient Engagement (PE) scale has been used to assess engagement in web-based programs in the medical setting [38,39]. The PE scale assesses engagement in the domains of caring for one’s health, concerns addressed about LCS, understanding LCS guidelines, and understanding information about LCS. Scores on the PE scale range from 1=low engagement to 5=high engagement.

To better interpret the results of the quantitative measures, we used qualitative approaches, including observations of the testing sessions and brief interviews at the completion of the testing sessions. These qualitative approaches have been applied in usability testing to identify barriers to the effective navigation of tools and processing of information [40,41]. The research assistants (RAs) who obtained the qualitative data underwent formal training in qualitative interviewing before the study. This process included 8 hours of training in qualitative methods in the Mixed Methods Research Lab at the University of Pennsylvania for our senior RAs (JP: Bachelors of Science with Major in Biology; JI: Bachelor of Science with a major in Neuroscience). Our junior RA was trained by the senior RAs on the observation of interviews and feedback (JM: Bachelor of Arts with a major in Psychology).

### Phase 1 Prototype Usability Testing

#### Phase 1 Study Participants and Recruitment

Veterans were eligible if they were aged 55 to 80 years, had a 30 pack-year history of cigarette smoking, and continued smoking sometime within the past 15 years. The exclusion criteria were cognitive impairment; a life expectancy of <2 years, as determined by their primary care provider; and having received LCS within the past 18 months. The participating sites were the Michael J Crescenz VA Medical Center in Philadelphia, Pennsylvania, and the West Haven VA Medical Center in West Haven, Connecticut.

We used the Corporate Data Warehouse to identify eligible veterans based on age, an algorithm of indication of tobacco use based on primary care visits and dental visit structured fields in the computer record, and pharmacy records of the use of smoking cessation medications, as well as having an upcoming primary care appointment. We sent recruitment letters with follow-up phone calls to confirm eligibility. Veterans who were interested were then scheduled for a study visit. Recruitment for the phase 1 protocol study occurred between August 2018 and January 2019. A voucher for US $50 was offered to compensate the participants for their time. The recruitment goal for veterans was 16 from each site (total of 32)—a sample size that has been validated in the literature for usability testing [42,43].

#### Phase 1 Prototype Usability Testing Procedures

Following informed consent and the completion of a baseline survey to assess demographic information, participants were individually seated in a room with an RA. Participants were provided with a tablet device to use the LCSDecTool. The session was audio recorded and transcribed. Following the use of each section of the tool (Table 2), the RA asked the participants to describe their user experience. The RA documented field notes during the session to highlight responses to specific sections. At the completion of the tool, the participants completed the SUS and EUCS scales [27,29].

**Table 2:** Qualitative feedback in phase 1 prototype usability testing.

Features and content of the LCSDecTool^a^		Feedback from phase 1 prototype testing	
		Veterans	Clinicians
**Computer based**			
	Accessed by a URL link on devices: tablet, desktop, laptop, and smartphone	Users varied in preferred device: tablet, laptop, phone	—^b^
**Overview of LCS^c^: simulated discussion between patient and provider**			
	Simulated dialog with questions and answers about LCS	Users found this engaging Most recognized that it was a physician and patient discussion and found this engaging The scrolling function was intuitive to most Recognized the format as similar to texting Easy to navigate One did not realize it was a physician–patient discussion	Consider adding audio Clarify who is speaking Dialog seems natural Shorten Define CT^d^ scan Change *Nodule* to *Spot* Liked clarification that a false positive is not a mistake Change *Doctor* to *Provider* Indicate most nodules are small
**Overview of LCS: clickable knowledge boxes**			
	6 knowledge boxes, each covering a key LCS content area; one must click on all boxes before advancing to the tool	Most found this to be more informative and easier to navigate than simulated dialog Some noted that the repetition of some content in this format reinforced the information that was being conveyed The pictures on each box were engaging	Add a box for *what is a CT scan?* Navigation may be confusing Symbols would be better than pictures Be careful about using relative risk reduction for mortality benefit Consider the pictorial representation of statistics Add a box for *what happens if my scan is abnormal?* Agree with bringing up annual screening; include that interval cancers may occur
**Pictograph**			
	Main outcomes from the National Lung Screening Trial displayed in pictograph: lung cancer deaths and deaths averted, false positives, biopsies, and complications	Users (except for 1) understood that the 2 side-by-side pictographs were comparing outcomes between screened and not screened populations Understood dots to represent people and colored dots to represent outcomes Some needed to be guided through the pictograph to understand	Good color contrast Would use with patients Helpful visual aid Describe a major complication Clarify screened and unscreened groups
**Value elicitation—rating scale 1 and rating scale 2**			
	Rating scale 1 response scale: much less likely to much more likely to want screening; rating scale 2 response scale: not at all concerned to extremely concerned	For most users, rating scale 2 was easier to use and demonstrated greater variation in ratings among benefits and potential harms of screening. One user found rating scale 1 to be more relevant and helpful in evaluating these attributes	Less user friendly than the attitudes section Shorten Explain that answers go to the summary page Lacks assessment of cost Prefers scale 2 Carry over stem to each question
**Cancer screening attitudes—rating scale**			
	An assessment of general cancer screening attitudes and beliefs	Questions were intuitive and easy to answer	Reads well Clarify what *repeat testing* means Clarify why these questions were asked
**Veteran-centric content—smoking cessation and mental health**			
	VA^e^ resources highlighted with the option to request a consultation	Most acknowledged that these were important, and some clicked boxes to request consultations. One user cautioned that raising the issue of anxiety may discourage a veteran from LCS	Provide phone numbers in a handout Note that the risk of lung cancer decreases after smoking cessation Change the description to *mental health or behavioral health provider* It is important to include smoking cessation to emphasize benefit, even with LCS State that smoking cessation is more effective than LCS in preventing lung cancer deaths Loved mental health access Include information specific to veterans Integrates well with the tool
**Enter questions for the provider**			
	Free text option; questions inserted on the summary sheet	Users all supported this feature	—^b^
**Summary page**			
	Includes ratings of values or attitudes; able to print, save, or email page	Users all supported this feature	Clarify where the email goes Simplify and shorten Title value responses with “Why I want screening” Clarify that it goes to the provider Improve that format of presenting scale results; use color coding Give suggestions to providers about how to address concerns; goal to distinguish beliefs from misunderstandings Like how it looks; will be helpful to providers
**Clinician portal**			
	Link from entry page to features for use at the point of care: pictograph and value and attitude assessment	—	Name *Clinician* or *Provider* rather than *Physician* portal Add picture with active link to the portal Make more accessible to the clinician Use term save *document* versus *PDF* Like that the provider has quick access to patient summary

^a^LCSDecTool: lung cancer screening decision tool.

^b^No information emerged for this feature.

^c^LCS: lung cancer screening.

^d^CT: computed tomography.

^e^VA: Veteran Affairs.

#### Phase 1 Prototype Analytic Plan

##### Quantitative Analysis

Descriptive statistics were used to summarize the following outcomes: (1) time in minutes for completion of the tool, (2) participant characteristics, and (3) scores on the SUS and EUCS scales. Participant characteristics included sex, race, ethnicity, age, and education level.

##### Qualitative Analysis

Members of the research team (MS, JP, and JM) reviewed the transcripts of the user sessions with veterans and the field notes documented by the RAs from provider interviews. Comments were summarized for each section of the LCSDecTool to indicate feedback from veteran users and clinicians regarding the features and content of the LCSDecTool (Table 2).

### Phase 2 High-Fidelity Usability Testing

#### Phase 2 Study Participants and Recruitment

Usability testing of the high-fidelity version was conducted as part of a pilot type 1 hybrid effectiveness-implementation trial that compared the LCSDecTool with a control web-based intervention providing general education about cancer screening. The participating sites were the Michael J Crescenz VA Medical Center in Philadelphia, Pennsylvania, and the West Haven VA Medical Center in West Haven, Connecticut. Eligibility criteria for veteran participants were identical to the phase 1 prototype usability testing protocol with the following exceptions: (1) the additional exclusion criteria of a prior diagnosis of cancer (except for stable prostate cancer or nonmelanomatous skin cancer) and (2) the additional inclusion criteria that the veterans’ primary care provider consent to participate in the study. Recruitment for the phase 2 high-fidelity usability study occurred between March 2019 and February 2020. Participants received a US $50 voucher for the baseline visit, which included usability testing.

#### Phase 2 High-Fidelity Usability Testing Procedures

Following informed consent and the completion of a baseline survey, participants were given a tablet that was open to the first page of the LCSDecTool. Participants were instructed to navigate the tool on their own. The RA observed the session, took field notes to document observed difficulties in navigation, and answered the participants’ questions during the usability session. The time from when the participant started to interact with the tool until the summary page was reached (ending the session) was documented by the RA.

Upon completion of the use of the tool, the RA conducted a brief interview with the participant, which included the following questions:

1. What were your general impressions of the LCSDecTool?
2. Did you have any trouble using the tool, and if so, describe (if the RA had noticed any issues in navigating the tool that were not mentioned, they would prompt by adding “I noticed you had trouble with...”)?
3. Do you have suggestions for improvements?
4. On the page that has boxes with knowledge content, do you think that it should be required to click on every box to move forward?
5. Did you find the tool repetitive in any way?

When the user responded affirmatively, follow-up questions were asked so that the patient could further elucidate.

Upon the completion of the usability testing session, participants proceeded to their scheduled primary care appointments. A postclinic survey was completed directly after the primary care appointment. The postclinic visit survey included the SUS, EUCS, and PE scales. The study was approved by the institutional review boards of the participating sites.

#### Phase 2 High-Fidelity Analytic Plan

##### Phase 2 Quantitative Analysis

Descriptive statistics were used to summarize participant characteristics, including sex, race, ethnicity, age, annual household income, and comorbidity. Descriptive statistics were used to summarize the responses to the SUS, EUCS, and PE scales.

##### Phase 2 Qualitative Analysis

We used a thematic analysis to analyze the qualitative data from the following sources: (1) notes taken by the RA while observing usability testing and (2) documented responses to the brief interview that followed the user session. Thematic analysis is an approach for identifying, analyzing, and reporting themes or patterns within a set of data using standard methods for qualitative research [44]. We used an inductive approach to identify themes pertaining to the user experience with the tool. A total of 2 members of the study team (JP and JM) initially reviewed the data and created an initial coding scheme. The data and codes were reviewed and finalized with input from additional members of the study team (MS and DK). Final coding was conducted by 2 independent coders, with differences resolved by consensus.

### Ethics Approval and Informed Consent

The study was approved by the institutional review board of the participating sites. Participants provided informed consent to complete the baseline survey in phase 1, and the clinicians of participating veterans signed informed consent forms in phase 2 at the time of enrollment in the study. IRB approval obtained from the Michael J Crescenz VA Medical Center, Philadelphia VA (IRB# 01635, IRB #01721, IRB#01780) and the VA Connecticut Healthcare System (MIRB# 02071, MIRB#02240).

## Results

### Phase 1 Prototype Usability Testing

#### Phase 1 Study Participants

A total of 70 recruitment letters were mailed to the Philadelphia VA Medical Center. Of the 70 veterans, 12 (17%) had confirmed eligibility by phone interviews and were enrolled in the study. A total of 101 letters were mailed to the West Haven VA Medical Center. Of these 101 veterans, 6 (5.9%) were confirmed to be eligible and were enrolled in the study. Of the 18 participants, 15 (83%) were male, 8 (44%) were African American, and 15 (83%) had up to a high school–level education. Additional participant characteristics are presented in Table 3.

**Table 3:** Description of study populations.

Participant characteristic		Prototype cohort^a^ (n=18)	High-fidelity cohort (n=43)
**Sex, n (%)**			
	Male	15 (83)	39 (91)
	Female	3 (17)	4 (9)
**Race, n (%)**			
	African American or Black	8 (44)	27 (63)
	Asian	—^b^	—
	Hawaiian native or Pacific Islander	—	—
	Native American or Alaska Native	—	—
	White	8 (44)	15 (35)
	Other	1 (6)^c^	—
	Unknown	2 (11)	1 (2)
**Ethnicity, n (%)**			
	Hispanic	2 (11)	1 (2)
	Non-Hispanic	16 (89)	42 (98)
Age (years), mean (SD)		64.7 (5.0)	64.5 (4.7)
**Education, n (%)**			
	Grade school	1 (6)	3 (7)
	Up to grade school	6 (33)	17 (40)
	High school or GED^d^	8 (44)	21 (49)
	Some college or university	3 (17)	1 (2)
	≥4 years of college	—^e^	1 (2)
**Annual household income (US $), n (%)**			
	0 to 25,000	—^a^	21 (49)
	>25,000 to 50,000	—^a^	12 (28)
	>50,000 to 75,000	—^a^	5 (12)
	>75,000 to 100,000	—^a^	1 (2)
	>100,000	—^a^	2 (5)
	Prefer not to answer	—^a^	2 (5)
**Comorbidity, n (%)**			
	Posttraumatic stress disorder	—^a^	20 (47)
	Depression	—^a^	17 (40)
	Arthritis	—^a^	17 (40)
	Asthma	—^a^	17 (40)
	Hypertension	—^a^	15 (35)
	Anxiety	—^a^	12 (28)
	Diabetes	—^a^	11 (26)
	Emphysema	—^a^	8 (19)
	Heart disease	—^a^	4 (9)
	Other	—^a^	4 (9)

^a^The prototype cohort did not have an assessment of income or comorbidity.

^b^There were no self-reports of race in these categories.

^c^One participant selected *Other* and *White*.

^d^GED: General Educational Development.

^e^No self-reports of education in this category.

#### Phase 1 Prototype Quantitative Results

In the phase 1 prototype usability testing, the mean of the SUS score (potential range: 0-100) was 81.90 (SD 9.80), corresponding to an excellent level of usability. The mean of the EUCS score (potential range 1-5) was 4.30 (SD 0.71; Table 4).

**Table 4:** Quantitative outcomes for phase 1 prototype and phase 2 high-fidelity usability.

Categorization				Prototype cohort (n=18), mean (SD)		High-fidelity cohort (n=43), mean (SD)
**SUS^a,b^: total (0-100); individual items (0-10)**						
	Total		81.90 (9.80)		65.76 (15.23)	
	I think I would like to use this tool frequently.		7.64 (2.18)		7.09 (2.11)	
	I found the tool unnecessarily complex.		8.75 (2.46)		7.09 (2.11)	
	I thought the tool was easy to use.		8.47 (152)		6.40 (2.45)	
	I think that I would need the support of a technical person to be able to use this tool.		8.06 (2.79)		7.03 (2.33)	
	I found the various functions in this tool were well integrated.		8.75 (1.29)		6.91 (2.17)	
	I thought there was too much inconsistency in this tool.		8.75 (1.96)		6.57 (2.25)	
	I would imagine that most people would learn to use this tool very quickly.		7.92 (2.46)		7.26 (1.79)	
	I found this tool very cumbersome to use.		7.92 (3.12)		6.22 (2.52)	
	I felt very confident using the tool.		8.47 (1.94)		7.44 (1.87)	
	I needed to learn a lot of things before I could get going with this tool.		7.22 (3.31)		5.11 (2.67)	
**EUCS^c,d^ measure (score 1-5)**						
	Total		4.30 (0.71)		3.91 (0.95)	
	**EUCS content subscale**		4.25 (0.90)		3.81 (0.93)	
		Does the web tool provide the precise information you need?	4.17 (0.99)		3.81 (1.11)	
		Does the web tool information content meet your needs?	4.28 (0.89)		3.74 (1.03)	
		Does the web tool provide help that seemed to be just about exactly what you need?	4.22 (01.17)		3.67 (1.06)	
		Did the web tool provide sufficient information?	4.33 (0.84)		4.02 (1.01)	
	**EUCS accuracy subscale**		4.25 (0.83)		3.87 (1.10)	
		Was the web tool accurate?	4.22 (0.88)		3.86 (1.10)	
		Were you satisfied with the accuracy of the web tool?	4.28 (0.89)		4.05 (0.95)	
	**EUCS format subscale**		4.28 (0.69)		3.97 (1.00)	
		Did you think the web tool information is presented in a useful manner?	4.28 (1.02)		4.05 (0.95)	
		Was the web tool information clear?	4.28 (0.83)		4.05 (1.05)	
	**EUCS ease of use subscale**		4.53 (0.70)		4.0 (1.02)	
		Was the web tool user friendly?	4.5 (0.86)		4.05 (0.10)	
		Was the web tool easy to use?	4.56 (0.62)		3.95 (1.13)	
	**EUCS timeliness subscale**		4.25 (0.83)		3.88 (1.06)	
		Did you get the web tool information you needed quickly?	4.28 (0.96)		3.86 (1.08)	
		Did the web tool provide up-to-date information?	4.22 (0.88)		3.91 (1.11)	
**PE^e,f^ tool (score 1-5)**						
	Total score		—^g^		4.12 (0.67)	
	How well did the tool support you in caring for your health?		—		4.00 (0.70)	
	How well were your concerns about lung cancer screening addressed?		—		4.27 (0.77)	
	How well did you understand the guidelines for lung cancer screening?		—		4.12 (0.76)	
	How well did you understand the information provided about lung cancer screening?		—		4.14 (0.83)	

^a^SUS: System Usability Scale.

^b^The SUS is a 10-item Likert scale with individual item scores ranging from 0 (low usability) to 10 (high usability) and a total score ranging from 0 to 100.

^c^EUCS: End User Computing Satisfaction.

^d^The EUCS is a 12-item scale measuring domains of content, accuracy, format, ease of use, and timeliness.

^e^PE: Patient Engagement.

^f^The PE scale includes four items assessing whether the tool (1) supports users in caring for their health, (2) addresses health concerns, (3) informs users about lung cancer screening guidelines, and (4) informs users about lung cancer screening. Scores on the PE scale range from 1 (low engagement) to 5 (high engagement).

^g^Patient Engagement was not assessed on the prototype cohort.

#### Phase 1 Prototype Qualitative Results

The veterans’ and clinicians’ qualitative feedback for the prototype testing are summarized in Table 2. Key feedback received from veterans included the following: (1) the dialog feature was engaging; (2) the knowledge boxes were an effective way of presenting information and reinforcing content introduced in the dialog feature; (3) the pictograph effectively conveyed a comparison of outcomes among screened versus nonscreened groups; (4) the value elicitation questions using a response scale measuring level of concern regarding potential harms were more intuitive to most than the response scale assessing if the attribute made it more or less likely for the veteran to have LCS; and (5) there was enthusiasm among users about the interactive features, including a text box and summary sheet to ask questions and share questions, values, and preferences with their provider. Clinician feedback included recommendations for simplifying the terminology used and enthusiasm for the pictograph as a visual aid to support provider–patient communication about LCS.

#### Modifications Made in the High-Fidelity Version

**Figure 1:**
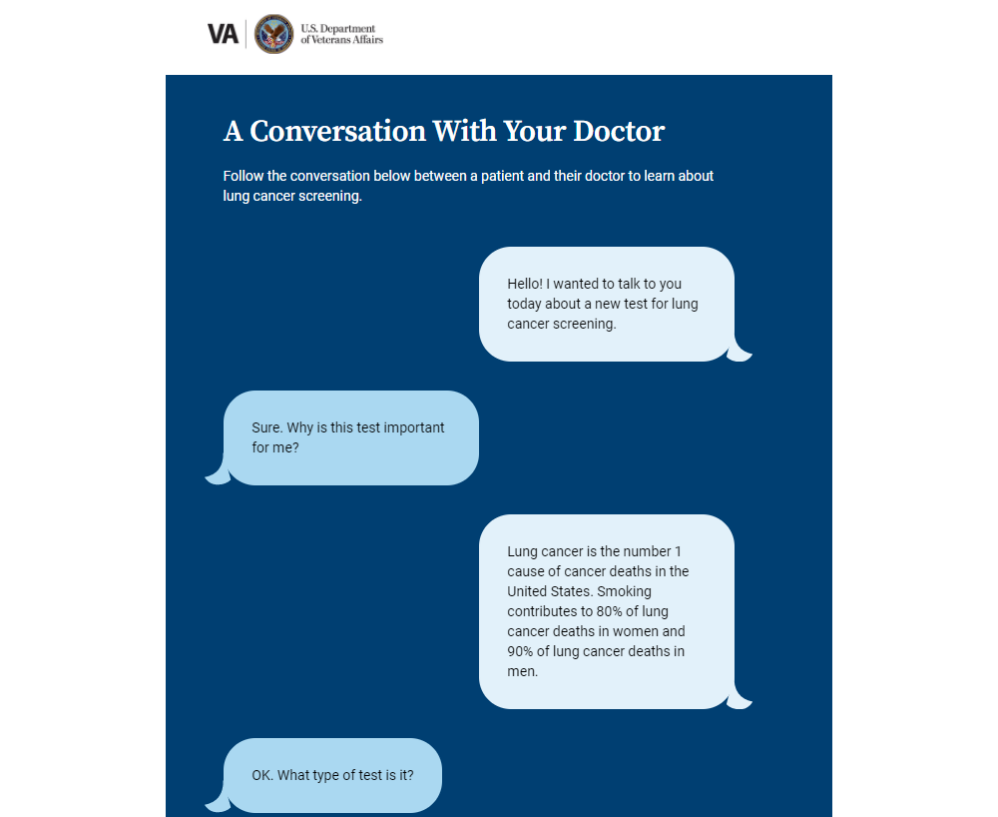
Illustration of the dialog feature in the lung cancer screening decision tool (LCSDecTool).

**Figure 2:**
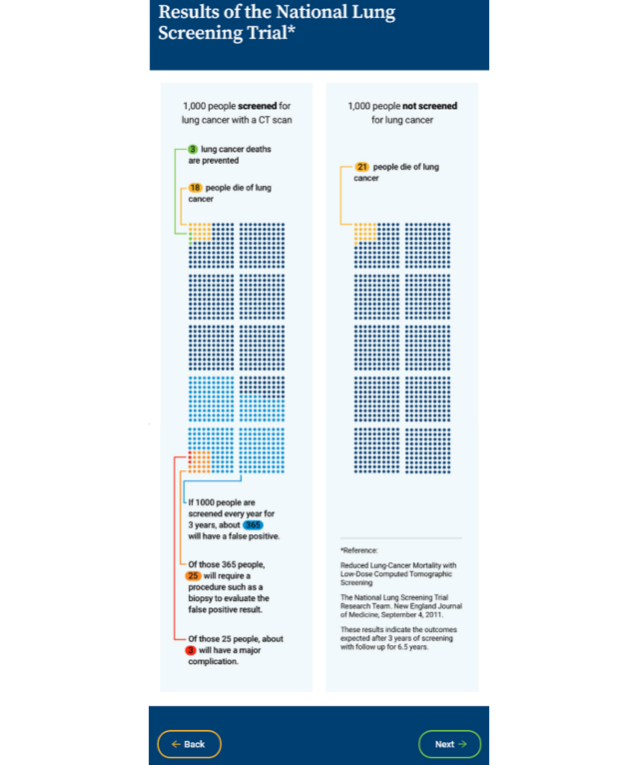
Illustration of the pictograph feature in the lung cancer screening decision tool (LCSDecTool). CT: computed tomography.

**Figure 3:**
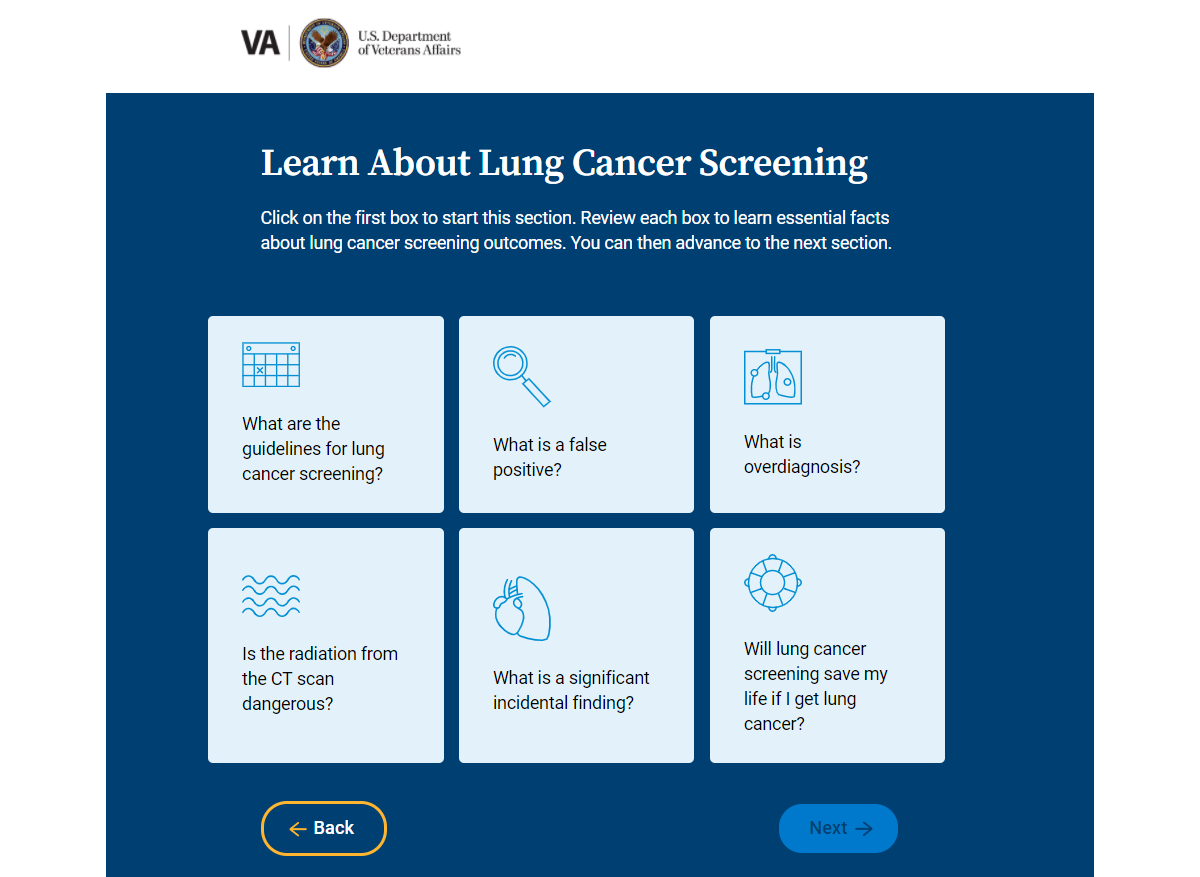
Illustration of the knowledge box feature in the lung cancer screening decision tool (LCSDecTool).

**Figure 4:**
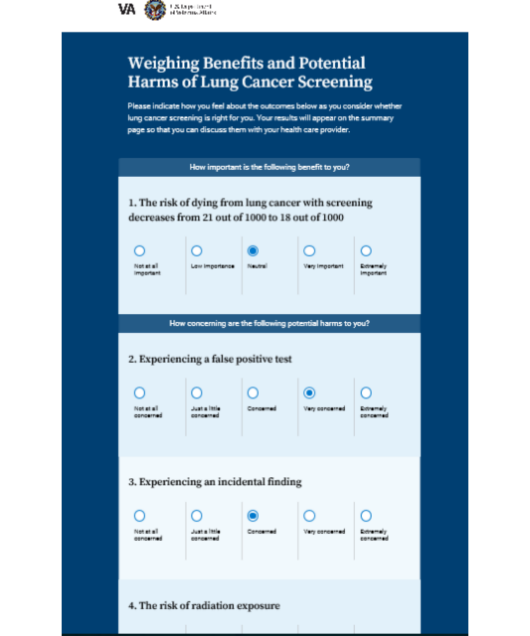
Illustration of the value elicitation feature of the lung cancer screening decision tool (LCSDecTool).

Results of the phase 1 testing informed changes made to the prototype in the development of the high-fidelity version, including the following: (1) use of a color scheme aligned with the US Department of Veterans Affairs branding, (2) replacement of stock graphics with icons and symbols, (3) use of directions and well-placed buttons to improve self-navigation, (4) a more prominent link from the entry page to the clinician portal, (5) simplified text and definitions, (6) single value clarification exercise, and (7) improved graphics and format of summary page to increase the visual impact and clarity. A link to the LCSDecTool is found in Multimedia Appendix 2. The dialog, knowledge box, pictograph, and value elicitation features are illustrated in Figures 1-4.

### Phase 2 High-Fidelity Testing

#### Phase 2 Study Participants

At the Philadelphia VA Medical Center, 465 recruitment letters were sent. Of these 465 persons, 376 (80.9%) were reached by phone, and 281 (60.4%) were confirmed to be eligible for the study. Of the 281 eligible persons, 80 (28.5%) were enrolled in the study, with 42 (53%) in the experimental arm and participating in the usability analysis. At the West Haven VA Medical Center, 136 letters were sent. Of these 136 individuals, 96 (70.6%) were reached by phone and found to be eligible for the study. Of the 96 eligible persons, 5 (5%) were enrolled in the study, with 1 (20%) in the experimental arm and participating in the usability analysis. Of the 43 participants, 27 (63%) were African American or Black, 39 (91%) were male, and 41 (95%) had up to a high school–level education. Additional demographic details are presented in Table 2.

#### Phase 2 High-Fidelity Quantitative Results

In the phase 2 high-fidelity usability testing, the mean SUS score was 65.76 (SD 15.23), corresponding to a good level of usability. The mean of the EUCS score (potential range 1-5) was 3.91 (SD 0.95). The mean PE score (potential range 1-5) was 4.12 (SD 0.67; Table 3). The median time to completion in minutes was 13 (IQR 7-30; Table 4).

#### Phase 2 High-Fidelity Qualitative Results

A total of five themes related to usability emerged from the qualitative analysis of the field note–documented observations and responses to the short interview. These themes were as follows: (1) a low baseline level of awareness and knowledge about LCS increased after using the LCSDecTool; (2) users sought more detailed descriptions about the LCS process; (3) the LCSDecTool was generally easy to use, but specific navigation challenges remained; (4) some noted difficulty understanding medical terms used in the LCSDecTool; and (5) the LCSDecTool evoked veteran struggles with prior attempts at smoking cessation (Textbox 1).

**Qualitative feedback from phase 2: high-fidelity usability testing.**

**Qualitative feedback from phase 2**

**Thematic analysis**

- Theme 1: low baseline awareness and knowledge about lung cancer screening (LCS) that increased after use of the LCS decision support tool (LCSDecTool)
- Theme 2: users sought more detailed descriptions about the LCS process
- Theme 3: the LCSDecTool was generally easy to use; however, specific navigation challenges remained
- Theme 4: some noted difficulty understanding medical terms used in the LCSDecTool
- Theme 5: the LCSDecTool evoked veteran struggles with prior efforts at smoking cessation

**Navigation challenges**

- Scrolling for physician–patient dialog (n=15)
- Advancing through knowledge boxes (n=10)

**Negative affective responses of using the tool**

- Worry about cancer risk
- Reading about the harms are scary
- Difficulty of smoking cessation

**Genuineness of tool**

- Dialog seemed scripted (n=1)

**Ease of understanding**

- Needed help to understand pictograph (n=1)

**Veteran-specific features**

- Resources for smoking cessation: some veterans were already familiar with the resources
- Mental health consultation: comment that a mention of anxiety related to LCS would discourage veterans from ever having LCS (n=1)

Theme 1 indicates a need for more information about LCS among this population. A participant stated that they “learned a lot and didn’t know much about LCS and lung cancer before using the tool.” Another stated that they “didn’t know that the VA even had a screening test.” This feedback supports our finding of high PE scores in the quantitative testing. Theme 2 reflects the desire for more information about the LCS process, with one of the participants asking, “What actually is a CT scan?” and another questioning, “if it is painful?” This theme indicates what information could be added to the tool to increase the content domain of the EUCS measure. Theme 3 indicates that the tool was generally easy to use; however, navigation was challenging for some of the features. For example, a participant described the tool as “helpful, with a lot of information, easy to use.” However, specific navigation problems were identified. A participant indicated that they “did not know how to scroll through the Dialogue,” and an RA observed and commented that another user was “stuck on Box page until I told him he had to click on the boxes.” Some participants had difficulty using the radio buttons on the value elicitation feature. Theme 4 indicates that some users struggled to understand the medical terminology. A participant stated that “some wording can get you twisted up” and provided feedback to “keep it simple and use plain language.” For example, the meaning of the words “nodule,” “CT Scan,” and “Overdiagnosis” were not understood by some users. This theme is relevant for interpreting the SUS scores in the format domain.

Theme 5 indicates that the tool evokes veterans’ struggles with prior attempts at smoking cessation. One of the specific veteran-centric features included in the tool highlighted the importance of smoking cessation and mental health. Users could click a radio button to request to speak with a primary care provider about smoking cessation or a mental health provider about either smoking cessation or LCS. Some users stated that they were already familiar with these resources or had successfully quit smoking. Others commented on the difficulty of smoking cessation, recalling multiple efforts to do so. A participant commented that he quit smoking for 1 year but recently restarted, stating, “don’t ask me why I started again, I don’t have a reason, I just did it,” and another commented, “I have tried the smoking cessation classes here at the VA but they don’t work.” Other users clicked the boxes conveying an interest in speaking about these topics to a provider.

## Discussion

### Principal Findings

The LCSDecTool was designed to create an engaging experience, inform veterans about LCS, support a value-aligned decision, and facilitate communication with their provider about LCS. We found that usability among veterans was good when administered in the context of a primary care clinical visit. These results provide evidence that an older group of US veterans can navigate tablet-based DAs. Our study was noteworthy for engaging veterans in the design of a decision support intervention in a meaningful way. In creating a veteran-centric tool, we involved veterans at every step of development and incorporated their feedback in an iterative process of tool development. Veterans were able to help determine the components of the SDM process and provide reactions and comments that could help other veterans. The motivation to provide feedback to help other veterans reflects a military culture of caring for and deriving satisfaction from helping other veterans [45].

Given that veterans in this age group may not have been familiar with technology, usability testing was particularly important to ensure that they could interact with and understand the information in the tool. Our usability findings were strengthened by conducting the assessment in the setting of a primary care visit where the LCSDecTool was designed to be used. Through this evaluation, we identified areas of strength and areas that require further refinement and modification.

The qualitative feedback obtained from our findings revealed 5 underlying themes. Two of these themes (low baseline awareness and knowledge about LCS that increased after use of the tool and the desire for more detailed descriptions of the LCS process) indicate that usability testing increased awareness of LCS. This is a key step in the process of adopting this evidence-based and provides preliminary evidence that the tool will increase awareness of LCS. Two additional themes (the tool was generally easier to use, but navigation challenges remain and difficulties in understanding some medical terminology remained) indicate the importance of veteran feedback in creating a tool that veterans are able to understand and use. These themes will guide further refinements of the tool. A final theme indicates that the tool may enhance discussions with providers about smoking cessation and mental health. This theme reinforces that veterans perceive a relationship between mental health, smoking cessation, and LCS and further supports the decision to address this relationship in the LCSDecTool.

Our study sample was a particularly vulnerable population in terms of sociodemographic factors (low level of education and income) and health status. Among those in the phase 2 high-fidelity cohort, 47% (20 out of 43) reported a diagnosis of PTSD. Rates of PTSD are known to be higher in veterans than in the general US population. Furthermore, among veterans, the rates of PTSD are higher in those receiving VA care than those who do not [46]. Veterans who receive care in the VA are also known to have lower education and income than veterans receiving care outside of the VA. Given these differences in socioeconomic status and health status, usability testing among a sample of veterans receiving VA medical care focuses our study on a more vulnerable population of veterans.

We observed a decrease in the usability measures from the prototype testing cohort to the high-fidelity testing cohort. There are several potential reasons for these differences. First, in the prototype cohort assessment, quantitative usability measures were collected immediately after completing the tool. In contrast, in the high-fidelity cohort, the user experience and quantitative usability assessment were separated by a clinic visit. The goal of this study was to assess the usefulness of the tool when used in the clinic setting. However, the intervening clinic visit may have decreased the salience of user experience. Of note, the purpose of the primary care visit was not limited to the topic of LCS, although addressing age and risk factors for appropriate cancer screening is an expected component of a primary care visit. Second, the study directed RAs to be more engaged with participants in the earlier prototype testing than in the high-fidelity testing. For example, in the prototype testing, the RAs discussed the participants’ experience with each section of the tool before progressing to the next section. The increased level of RA engagement during the prototype user session may have positively affected user experience.

According to the TAM, users need to perceive the technology as useful and easy to use to continue using it. Our qualitative data provide additional insights on participant perceptions of the tool regarding usability and usefulness. The design of our value assessment measures was based on qualitative studies conducted with veterans regarding how they perceived and valued the potential benefits and harms of LCS [30]. In usability testing, the value assessments were completed without difficulty, with some participants commenting that they enjoyed responding to these scales and found them helpful. Our PE survey indicates that users of the LCSDecTool felt engaged in the decision-making process, a primary goal of any SDM intervention, as in the development of other eHealth applications [35,36]. Our results suggest that users perceived that the tool supported them in caring for their health, addressed their concerns, and informed them about LCS.

### Comparison With Other Work

In our study, we observed that 100% of the participants completed the use of the LCSDecTool. Completing the tool included moving through all sections, responding to the value assessment questions, and submitting the summary page. In a recent Cochrane review, which was a subgroup analysis of 105 studies involving >31,000 participants, the median effect of a DA on the length of a medical consultation was to lengthen the consultation by 2.6 minutes in comparison with usual care [24]. Although the length of the LCSDecTool did not emerge as a concern among users in our qualitative feedback, the time required to complete the tool could have contributed to lower scores on the SUS and EUCS scales. Our qualitative data suggest that improving navigation and allowing users to choose which sections to review would decrease the time to completion of the LCSDecTool without compromising usability and effectiveness.

Prior studies on cancer screening DAs provide a comparison of the usability scores for the LCSDecTool. Carter-Harris et al [8] developed an LCS DA that included audio and video features and scripts tailored to the user’s smoking status. The tool, named *Lung Talk*, reported mean SUS scores of 75.7 (SD 7.9), indicating a good level of usability [8]. Coe et al [47] developed a breast cancer screening DA for use among multi-ethnic women. This tool, named *Real Risks*, reported mean SUS scores of 80.0 (range 50.0-95.0) and 66.3 (range 55.0-75.0) for the English and Spanish versions, respectively [47]. Our study design is unique in reporting usability scores prospectively as the tool moved from the evaluation of a prototype to the evaluation of a high-fidelity version within a clinical setting. In our study, the SUS scores decreased when the tool was integrated into the clinical setting but remained at an acceptable level of usability.

Our LCSDecTool differs from existing LCS DAs in several respects. It was developed using the principles of user-centered design to increase engagement with the tool among a veteran population. This includes the look and feel of the tool, such as using the Department of Veterans Affairs branding, content that acknowledges the mental health conditions that may affect LCS, and smoking cessation referrals specific for veterans receiving care in VA medical centers.

### Limitations

Our study has some limitations. First, data for the phase 2 high-fidelity version were obtained in the context of a pilot hybrid type 1 effectiveness-implementation trial. In this research setting, participants were able to ask an RA for help with the tool if needed. This may not be feasible in routine care outside the context of a research study. Second, the LCSDecTool was tested on a limited number of veterans and clinical sites. Our population reports high rates of mental health conditions and other comorbidities and has particularly low levels of education and income. The findings may not be generalizable to a broader population of veterans or the general US population. Despite these limitations, the strengths of our study include the evaluation of the usability of the LCSDecTool among veterans who are diverse in race, meet eligibility criteria for LCS, and are receiving care in a VA primary care setting.

### Conclusions

In conclusion, our study found that a web-based LCS decision support tool developed for and tested among US veterans receiving care in a VA medical center demonstrates an acceptable level of usability. We designed the LCSDecTool for use before and during a clinical visit, incorporating content, formats, and functions that can be used across these settings. The decision regarding LCS requires patients and their providers to consider scientific evidence of benefits and harms, as well as patient values, priorities, and beliefs. Given these complexities, it is important to conduct usability testing of this patient-centered LCSDecTool in its target population. Our findings support the use of this eHealth technology in the primary care clinical setting as a way of engaging veterans, informing them about a new cancer control screening test, and preparing them to participate in an SDM discussion with their provider. Our study further indicates that involving veterans in all phases of the development of the tool extends veteran involvement to the design of SDM processes. Future work is needed to fully address the information and decision support tools that will help veterans understand and apply the principles of SDM for LCS in the context of a comprehensive health care program.

## Appendix

Multimedia Appendix 1 Prototype Lung Cancer Screening Tool Usability Assessment field notes form.

Multimedia Appendix 2 Link to the Lung Cancer Screening Decision Tool.

## Abbreviations

DA: decision aid
EUCS: End User Computing Satisfaction
LCS: lung cancer screening
LCSDecTool: LCS decision tool
NLST: National Lung Screening Trial
PARiHS: Promoting Action on Research Implementation in Health
PE: Patient Engagement
PTSD: posttraumatic stress disorder
RA: research assistant
SDM: shared decision-making
SUS: System Usability Scale
TAM: Technology Acceptance Model
VA: Veteran Affairs
