# Supplementary material for: A Veteran-Centric Web-Based Decision Aid for Lung Cancer Screening: Usability Analysis
Source: JMIR Form Res. 2022 Apr 8;6(4):e29039. doi: 10.2196/29039 (PMC9034418; doi:10.2196/29039)
Supplement: Multimedia Appendix 2 [file formative_v6i4e29039_app2.docx]

The LCSDecTool High Fidelity research version can be viewed at: <http://va-lung.punkave.net/> using the ID code of 0001.
